# GENERATING OLDER ACTIVE LIVES DIGITALLY (GOALD) THROUGH SPORT-BASED REMINISCENCE

**DOI:** 10.1093/geroni/igad104.2176

**Published:** 2023-12-21

**Authors:** Richard Haynes, John Ritchie, Simone Tomaz

**Affiliations:** University of Stirling, Stirling, Scotland, United Kingdom; Communications, Media and Culture, Stirling, Scotland, United Kingdom; University of Stirling, Stirling, Scotland, United Kingdom

## Abstract

How do digitally-delivered meaningful activities, such as sport-based reminiscence, influence older people’s health and well-being and intergenerational relationships? Developing and strengthening older people’s ’connectivities’ through their links with community, resources and meaningful activities is a key aspect of supporting healthy ageing and reducing health inequalities in later life. Sport-based reminiscence has become an established activity to facilitate social connectivity and increasingly, digital connectivity, which can support an individual’s sense of health and well-being. This paper reports on research undertaken within a three-year research programme on ’Connectivity and Digital Design for Promoting Health and Well-being Across Generations, Places and Spaces’ focusing on research conducted with four community-based co-production groups in Scotland. The qualitative research was developed with a stakeholder advisory group and co-production groups in care homes and in community. Data was gathered from recording of meetings and researcher observations thematically analysed to document shared experiences of digitally enabled sport reminiscence over a ten month period. We report on the efficacy of live-streamed and digital sport heritage experiences for triggering reminiscence in a hybrid context. We emphasise the need for structured facilitation of hybrid online/in-person reminiscence, which can clearly produce meaningful and enjoyable experiences for older people, and building connectivity.

